# Supplementary material for: The interaction between endogenous GABA, functional connectivity, and behavioral flexibility is critically altered with advanced age
Source: Commun Biol. 2022 May 6;5:426. doi: 10.1038/s42003-022-03378-w (PMC9076638; doi:10.1038/s42003-022-03378-w)
Supplement: Supplementary file 2 — Supplementary Information [file 42003_2022_3378_MOESM2_ESM.pdf]

Supplementary Material for
The interaction between endogenous GABA, functional connectivity and behavioral flexibility is critically altered with advanced age

Kirstin-Friederike Heise[1,2*], Laura Rueda-Delgado[1,3], Sima Chalavi[1,2], Bradley R. King[1,2,4], Thiago Santos Monteiro[1,2], Richard A. E. Edden[5,6], Dante Mantini[1,7], Stephan P. Swinnen[1,2]

1 Department of Movement Sciences, Movement Control and Neuroplasticity Research Group, KU Leuven, Leuven, Belgium
2 KU Leuven Brain Institute, Leuven, Belgium
3 School of Psychology, Trinity College Dublin, Dublin 2, Ireland
4 Department of Health & Kinesiology, College of Health, University of Utah, Salt Lake City, Utah, USA
5 The Russell H. Morgan Department of Radiology and Radiological Science, The Johns Hopkins University School of Medicine, Baltimore, MD, USA
6 F. M. Kirby Research Center for Functional Brain Imaging, Kennedy Krieger Institute, Baltimore, MD, USA
7 Brain Imaging and Neural Dynamics Research Group, IRCCS San Camillo Hospital, Venice, Italy

[*]**Correspondence to**
Kirstin-Friederike Heise
Movement Control and Neuroplasticity Research Group
KU Leuven
Tervuurse Vest 101; 3001 Leuven, Belgium
Email: kirstin.heise@kuleuven.be

Index

SUPPLEMENTARY RESULTS FOR GABA+ DATA 3

SUPPLEMENTARY TABLE 1 DESCRIPTIVE STATISTICS OF GABA+ AND QUANTITATIVE QUALITY METRICS 3
SUPPLEMENTARY TABLE 2 ANALYSIS OF DEVIANCE TABLE FOR BACKWARDS SELECTION OF PARAMETERS PREDICTING GABA+ .4
SUPPLEMENTARY TABLE 3 RESULTS FOR GAMMA GLMM PREDICTING GABA+ (FINAL MODEL) 4
SUPPLEMENTARY TABLE 4 MODEL ESTIMATED MARGINAL MEANS CONTRASTS FOR EFFECT OF GROUP X VOXEL INTERACTION ON GABA+ LEVELS 6
SUPPLEMENTARY NOTE 1 DISCUSSION OF DECREASING GABA+ CONCENTRATION WITH HIGHER GM FRACTION IN THE OLDER ADULTS 6

SUPPLEMENTARY RESULTS FOR BEHAVIOURAL DATA 6

SUPPLEMENTARY TABLE 5 GROUP STATISTICS OF OUTCOME PARAMETERS OF THE BIMANUAL TRANSITION TASK 6
SUPPLEMENTARY TABLE 6 RESULTS FOR LOGISTIC GLMM PREDICTING FAILED TRANSITIONS [TRIALS WITH 100% ERROR RATE]6
SUPPLEMENTARY TABLE 7 RESULTS FOR LOGISTIC GLMM TO PREDICT FULLY CORRECT TRANSITIONS [TRIALS WITH 0% ERROR] 8
SUPPLEMENTARY TABLE 8 RESULTS FOR BETA GLMM TO PREDICT CUMULATIVE ERROR RATE [0<ERROR RATE/100 <1] 9
SUPPLEMENTARY TABLE 9 RESULTS FOR GAMMA GLMM TO PREDICT TRANSITION LATENCY 10
SUPPLEMENTARY NOTE 2 THUMB REACTION TIME (TRT) – OPERATIONALIZATION AND STATISTICAL ANALYSIS 11
SUPPLEMENTARY TABLE 10 RESULTS FOR GAMMA GLMM TO PREDICT THUMB REACTION TIME 11

SUPPLEMENTARY MATERIAL FOR THE EEG DATA ANALYSIS 12

SUPPLEMENTARY FIGURE 4 STATISTICAL RESULTS OF SPECTRAL POWER CHANGES. 12
SUPPLEMENTARY FIGURE 5 STIMULUS-LOCKED ANALYSIS OF ISPC MODULATION 13

SUPPLEMENTARY RESULTS FOR PHASE ANGLE DIFFERENCES 14

SUPPLEMENTARY FIGURE 1 – PHASE ANGLE DIFFERENCES POOLED OVER TRANSITION MODES WITHIN AGE GROUP 14
SUPPLEMENTARY NOTE 3 RAYLEIGH TEST FOR DISTRIBUTION OF PHASE ANGLE DIFFERENCES BETWEEN LEFT S/M1 AND RIGHT S/M1 SOURCES AT TIME OF TRANSITION FOR TRANSITION MODES POOLED OVER AGE GROUPS. 14
SUPPLEMENTARY TABLE 11 RAYLEIGH TEST FOR DISTRIBUTION OF PHASE ANGLE DIFFERENCES BETWEEN LEFT S/M1 AND RIGHT S/M1 SOURCES AT TIME OF TRANSITION ACCOUNTING FOR GABA+ CONCENTRATION RELATIVE TO WITHIN GROUP MEDIAN 14
SUPPLEMENTARY TABLE 12 RAYLEIGH TEST FOR DISTRIBUTION OF PHASE ANGLE DIFFERENCES BETWEEN LEFT S/M1 AND RIGHT S/M1 SOURCES AT BASELINE [START CUE – 300MS] ACCOUNTING FOR GABA+ CONCENTRATION RELATIVE TO WITHIN GROUP MEDIAN 15
SUPPLEMENTARY TABLE 13 2-WAY ANOVA TESTING GROUP (OLDER VS. YOUNG) X GABA(LOW VS. HIGH) FOR MEAN PHASE ANGLE DIFFERENCE BETWEEN LEFT S/M1 AND RIGHT S/M1 SOURCES AT BASELINE [START CUE – 300MS] 15
SUPPLEMENTARY TABLE 14 CIRCULAR-LINEAR CORRELATION PHASE ANGLE DIFFERENCE ~ SUBSEQUENT ERROR 15
SUPPLEMENTARY FIGURE 2 ASSOCIATION BETWEEN BAND-SPECIFIC LEFT - RIGHT S/M1 PHASE DIFFERENCE AT BASELINE [START CUE – 300MS] AND SUBSEQUENT PERFORMANCE POOLED OVER TRANSITION CONDITIONS (GROUP AVERAGE OF SINGLE TRIAL BASELINE, ERROR REPRESENTS SUBSEQUENT TRIAL FOLLOWING THE START CUE) 16
SUPPLEMENTARY TABLE 15 COMPARISON LEFT -RIGHT S/M1 VERSUS OCC-S/M1 16
SUPPLEMENTARY TABLE 16 2-WAY ANOVA TESTING MEAN DIRECTION CONNECT (LEFT S/M1 -RIGHT S/M1 VS. OCC-LEFT S/M1) X GROUP (OLDER VS YOUNG) 16
SUPPLEMENTARY TABLE 17 2-WAY ANOVA TESTING MEAN DIRECTION CONNECT (LEFT S/M1 - RIGHT S/M1 VS. OCC-RIGHT S/M1) X GROUP (OLDER VS YOUNG) 17
SUPPLEMENTARY TABLE 18 CIRCULAR-LINEAR CORRELATION PHASE ANGLE DIFFERENCE ~ SUBSEQUENT ERROR 17
SUPPLEMENTARY FIGURE 3 ASSOCIATION BETWEEN BAND-SPECIFIC OCC-M1 PHASE DIFFERENCE AT TIME OF TRANSITION AND SUBSEQUENT PERFORMANCE POOLED OVER TRANSITION CONDITIONS 18

SUPPLEMENTARY RESULTS FOR THE BAYESIAN MODERATED MEDIATION ANALYSES 19

SUPPLEMENTARY TABLE 19 REGRESSION COEFFICIENTS OF BAYESIAN MODERATED MEDIATION MODELS 19

SUPPLEMENTARY REFERENCES 20

Supplementary results for GABA+ data

Supplementary Table 1 Descriptive statistics of GABA+ and quantitative quality metrics

		LEFT S/M1			*RIGHT S/M1*			*OCC*		
	Metric	*mean*	*sd*	*range*	*mean*	*sd*	*range*	*mean*	*sd*	*range*
YOUNG	GABA+	2.517	0.244	0.91 (2.02-2.93)	2.389	0.241	1.17 (1.97-3.13)	2.859	0.23	0.82 (2.38-3.2)
	GABA SNR	31.438	5.323	21.26 (20.86-42.13)	28.976	3.914	13.82 (24.07-37.89)	23.945	5.142	23.4 (14.18-37.58)
	GABA Fit Error	3.564	0.802	3.04 (2.5-5.54)	3.94	0.69	2.77 (2.78-5.55)	3.861	0.58	2.48 (2.32-4.8)
	GABA FWHM	19.728	1.088	4.68 (17.14-21.82)	19.852	1.428	6.06 (15.95-22.01)	21.104	1.03	3.56 (19.39-22.95)
	NAA SNR	350.501	69.802	240.24 (245.16-485.4)	323.816	118.315	391.46 (109.77-501.23)	360.697	75.164	250.33 (273.97-524.3)
	Drift	0.39	0.118	0.5 (0.22-0.72)	0.37	0.087	0.3 (0.21-0.51)	0.559	0.187	0.59 (0.27-0.86)
	Frequency Offset	0.006	0.008	0.03 (-0.01-0.02)	0.017	0.013	0.05 (-0.01-0.04)	0.002	0.004	0.02 (0-0.01)
	NAA FWHM	9.342	1.386	4.58 (7.53-12.1)	8.429	1.222	5.13 (6.38-11.5)	9.917	1.302	5.84 (8.24-14.07)
	GM fraction	0.355	0.028	0.11 (0.3-0.41)	0.379	0.026	0.09 (0.33-0.43)	0.642	0.031	0.11 (0.59-0.7)
	WM fraction	0.562	0.03	0.12 (0.52-0.63)	0.523	0.028	0.1 (0.47-0.57)	0.271	0.032	0.13 (0.21-0.34)
	CSF fraction	0.083	0.019	0.07 (0.04-0.11)	0.098	0.019	0.07 (0.06-0.13)	0.087	0.018	0.06 (0.06-0.12)
OLDER	GABA+	2.325	0.247	1.11 (1.98-3.09)	2.197	0.323	1.08 (1.65-2.73)	2.851	0.391	1.92 (1.99-3.91)
	GABA SNR	25.612	3.556	11.17 (20.1-31.26)	23.437	3.638	15.17 (17.4-32.57)	20.139	4.13	18.39 (14.06-32.45)
	GABA Fit Error	3.985	0.69	2.67 (2.7-5.37)	4.268	1.983	8.44 (2.32-10.76)	4.488	1.426	5.83 (2.35-8.18)
	GABA FWHM	20.069	1.083	4.59 (18.71-23.3)	19.617	1.122	4.35 (17.98-22.33)	21.828	1.353	5.91 (17.74-23.65)
	NAA SNR	285.801	63.379	244.38 (155.31-399.69)	310.188	105.774	472.95 (126.56-599.51)	275.062	44.029	165.07 (192.78-357.84)
	Drift	0.632	0.308	1.39 (0.34-1.73)	0.677	0.236	0.9 (0.35-1.26)	0.816	0.268	0.79 (0.4-1.19)
	Frequency Offset	0.007	0.01	0.05 (-0.01-0.04)	0.018	0.015	0.07 (-0.02-0.05)	0.011	0.009	0.04 (0-0.04)
	NAA FWHM	9.647	1.012	4.05 (8.26-12.31)	8.789	1.04	4.03 (6.28-10.31)	10.114	1.226	4.7 (7.46-12.16)
	GM fraction	0.267	0.033	0.14 (0.2-0.34)	0.291	0.045	0.17 (0.2-0.38)	0.526	0.056	0.26 (0.36-0.62)
	WM fraction	0.591	0.056	0.24 (0.45-0.69)	0.555	0.056	0.23 (0.43-0.66)	0.307	0.027	0.1 (0.25-0.35)
	CSF fraction	0.143	0.05	0.22 (0.06-0.28)	0.154	0.049	0.18 (0.09-0.27)	0.168	0.059	0.26 (0.08-0.34)

SNR signal-to-noise ratio, FWHM full width half maximum, GM grey matter, WM white matter, CSF cerebrospinal fluid

Supplementary Table 2 Analysis of deviance table for backwards selection of parameters predicting GABA+

Parameter	X^2	*Df*	*p*
GROUP	13.467	1	.0002*
VOXEL	44.917	2	<.0001*
GABA SNR (centered)	5.092	1	.024*
GABA Fit Error (centered)	4.007	1	.045*
NAA SNR (centered)	0.199	1	.66
Drift (centered)	0.128	1	.72
Frequency Offset (centered)	17.857	1	<.0001*
NAA FWHM (centered)	0.238	1	.63
raw GM fraction (centered)	15.372	1	<.000*
GROUP × VOXEL	6.942	2	.031*
GROUP × GABA SNR (centered)	0.669	1	.413
VOXEL × GABA SNR (centered)	0.523	2	.77
GROUP × GABA Fit Error (centered)	0.408	1	.52
VOXEL × GABA Fit Error (centered)	9.315	2	.009*
GROUP × NAA SNR (centered)	0.079	1	.78
VOXEL × NAA SNR (centered)	0.51	2	.78
GROUP × Drift (centered)	0.491	1	.48
VOXEL × Drift (centered)	1.03	2	.60
GROUP × Frequency Offset (centered)	0.007	1	.93
VOXEL × Frequency Offset (centered)	1.141	2	.57
GROUP × NAA FWHM (centered)	0.154	1	.70
VOXEL × NAA FWHM (centered)	3.097	2	.21
GROUP × raw GM fraction (centered)	8.015	1	.005*
VOXEL × raw GM fraction (centered)	3.411	2	.18

Supplementary Table 3 Results for Gamma GLMM predicting GABA+ (Final Model)

Type II Wald statistics

Predictors	X^2	*df*	*p*
GROUP	15.175	1	<.0001
VOXEL	45.042	2	<.0001
GABA SNR (centered)	6.736	1	.009
GABA Fit Error (centered)	5.559	1	.018
raw GM fraction (centered)	15.363	1	<.0001
NAA SNR (centered)	0.13	1	.72
Drift (centered)	0.041	1	.84
Frequency Offset (centered)	17.197	1	<.0001
NAA FWHM (centered)	0.123	1	.73
GROUP × VOXEL	9.566	2	.008
VOXEL × GABA Fit Error (centered)	5.841	2	.054
GROUP × raw GM fraction (centered)	6.821	1	.009

Parameter estimates based on references categories indicated

Predictors	*Estimates (β)*	*std. Error*	*CI*	*Statistic (X^2)*	*p*

(Intercept)	2.855	0.264	2.337 – 3.373	10.799	**<.0001**
Drift (centered)	-0.005	0.027	-0.058 – 0.047	-0.203	0.84
Frequency Offset (centered)	-0.092	0.022	-0.135 – -0.048	-4.147	**<.0001**
GABA Fit Error (centered)	-0.122	0.038	-0.197 – -0.047	-3.202	**.001**
GABA SNR (centered)	0.077	0.030	0.019 – 0.135	2.595	**.009**
raw GM fraction (centered)	-0.031	0.154	-0.334 – 0.272	-0.201	.84
NAA FWHM (centered)	0.009	0.025	-0.039 – 0.057	0.351	.73
NAA SNR (centered)	-0.008	0.022	-0.052 – 0.036	-0.360	.72
GROUP [YOUNG]	*Reference*				
GROUP [OLDER] * raw GM fraction	-0.489	0.187	-0.856 – -0.122	-2.612	**.009**
GROUP [OLDER] * VOXEL [LEFT S/M1]	-1.190	0.388	-1.950 – -0.430	-3.070	**.002**
GROUP [OLDER] * VOXEL [RIGHT S/M1]	-1.096	0.356	-1.794 – -0.398	-3.076	**.002**
GROUP [OLDER]	0.549	0.282	-0.004 – 1.101	1.945	.052
VOXEL [OCC]	*Reference*				
VOXEL [LEFT S/M1] * GABA Fit Error (centered)	0.134	0.063	0.011 – 0.257	2.134	**.033**
VOXEL [LEFT S/M1]	-0.464	0.335	-1.121 – 0.193	-1.383	.17
VOXEL [RIGHT S/M1]	-0.468	0.309	-1.075 – 0.138	-1.514	.13
VOXEL [RIGHT S/M1] * GABA Fit Error (centered)	0.093	0.046	0.003 – 0.183	2.032	**.04**
**Random Effects**					
σ^2	0.01				
$\tau_{00\ subject}$	0.01				
ICC	0.68				
$N_{subject}$	44				
Observations	130				
Marginal R^2 / Conditional R^2	0.827 / 0.945				
AIC / BIC	-7.34 / 44.27				

Supplementary Table 4 Model estimated marginal means contrasts for effect of GROUP X VOXEL interaction on GABA+ levels

within group – voxel comparison

GROUP	VOXEL CONTRAST	Difference	SE	CI_low	CI_high	z	p_{holm}
OLDER	LEFT S/M1 -RIGHT S/M1	-0.09	0.065	-0.28	0.101	-1.382	0.52
	OCC - LEFT S/M1	1.654	0.227	0.987	2.32	7.285	**<.0001**
	OCC - RIGHT S/M1	1.564	0.215	0.933	2.196	7.271	**<.0001**
YOUNG	LEFT S/M1-RIGHT S/M1	0.005	0.071	-0.204	0.213	0.065	.95
	OCC - LEFT S/M1	0.464	0.335	-0.52	1.448	1.383	.52
	OCC - RIGHT S/M1	0.468	0.309	-0.44	1.377	1.514	.52

between group comparison

GROUP CONTRAST	VOXEL	Difference	SE	CI_low	CI_high	z	p_{holm}
YOUNG - OLDER	LEFT S/M1	0.641	0.146	0.213	1.07	4.396	**<.0001**
	RIGHT S/M1	0.547	0.122	0.189	0.906	4.48	**<.0001**
	OCC	-0.549	0.282	-1.377	0.279	-1.945	.26

Supplementary Note 1 Discussion of decreasing GABA+ concentration with higher GM fraction in the older adults

Including GM fraction into the statistical model in addition to employing a tissue-correction method allowed us to directly investigate the effect of varying GM fraction on the GABA+ concentration in the voxels of interest between the two age groups. Our results suggest that while GM atrophy is present in the older as compared to the young participants, the reduction of GABA+ concentration does not parallel the GM atrophy in the older. While one would intuitively assume decreasing GABA+ levels with reduced GM fraction, age-related structural and functional alterations might not necessarily follow a linear trend. Among others, an initial upregulation of GABA synthesis [1], changes in GABA reuptake and transporter activity[2], or increases in GABA receptor availability measured with PET[3] may go alongside with GM atrophy and lead at least transiently to increased GABA+ concentration as measured with MRS. These results require careful consideration in the application of standard GABA quantification methods in ageing populations.

Supplementary results for behavioural data

Supplementary Table 5 Group statistics of outcome parameters of the bimanual transition task

	Total transition	*Failed transitions*	*Fully correct transitions*	*Cumulative error rate [in %]*		*Transition latency [in ms]*	
GROUP	*N*	*N (% of total)*	*N (% of total)*	*median*	*ci*	*median*	*ci*
young	2562	86 (3.3)	102 (3.9)	14.19	0.57	610	10.68
older	2684	198 (7.8)	285 (10.6)	14.29	0.76	807	16.77

Supplementary Table 6 Results for logistic GLMM predicting failed transitions [trials with 100% error rate]

Type II Wald statistics

Predictors	X^2	*df*	*p*
GROUP	2.19	1	.14
TRANSITION MODE	34.99	1	<.0001
nTRIALSc	9.86	1	.002

Predictors	X^2	*df*	*p*
GROUP × TRANSITION MODE	1.78	1	.18
GROUP × nTRIALSc	2.63	1	.11
TRANSITION MODE × nTRIALSc	0.03	1	.85
GROUP × TRANSITION MODE × nTRIALSc	4.38	1	.04

Parameter estimates based on references categories indicated

Predictors	*Odds Ratios*	*std. Error*	*CI*	*Statistic (X^2)*	*p*
(Intercept)$	-5.66	0.597	-6.83 – -4.49	-9.48	**<.0001**
GROUP[young]	*Reference*				
TRANSITION MODEAP × nTRIALSc	1.586	0.411	0.954 – 2.635	1.78	.08
GROUP[older]	2.315	1.772	0.517 – 10.378	1.10	.27
nTRIALSc	0.699	0.141	0.471 – 1.037	-1.78	.08
TRANSITION MODE[into IP]	*Reference*				
GROUP[older] × TRANSITION MODE[into AP]	1.427	0.472	0.746 – 2.729	1.08	.28
GROUPolder × TRANSITION MODE[into AP] × nTRIALSc	0.506	0.165	0.267 – 0.957	-2.10	**.036**
GROUPolder × nTRIALSc	1.163	0.294	0.709 – 1.908	0.60	.55
TRANSITION MODE[into AP]	2.075	0.542	1.244 – 3.462	2.80	**.005**

**Random Effects**	
σ^2	3.29
$\tau_{00\ subjID}$	4.20
ICC	0.56
N $_{subjID}$	42
Observations	5124
Marginal R^2 / Conditional R^2	0.070 / 0.592
AIC / BIC	1329.3 / 1388.1

[$] *Intercept given on log scale.*

Supplementary Table 7 Results for logistic GLMM to predict fully correct transitions [trials with 0% error]

Type II Wald statistics

Predictors	X^2	*df*	*p*
GROUP	15.426	1	<.0001
TRANSITION MODE	24.376	1	<.0001
nTRIALSc	0.046	1	.83
GROUP × TRANSITION MODE	1.291	1	.26
GROUP × nTRIALSc	0.567	1	.45
TRANSITION MODE × nTRIALSc	0.013	1	.91
GROUP × TRANSITION MODE × nTRIALSc	1.338	1	.25

Parameter estimates based on references categories indicated

Predictors	*Odds Ratios*	*std. Error*	*CI*	*Statistic (X^2)*	*p*
(Intercept)[$]	-3.26	0.277	-3.80 – -2.72	-11.77	**<.0001**
GROUP[young]	*Reference*				
TRANSITION MODE[into AP] × nTRIALSc	1.264	0.283	0.816 – 1.959	1.05	.29
GROUP[older]	3.520	1.246	1.758 – 7.046	3.55	**<.001**
nTRIALSc	0.979	0.124	0.764 – 1.254	-0.17	.87
TRANSITION MODE[into IP]	*Reference*				
GROUP[older] × TRANSITION MODE[into AP]	1.374	0.360	0.822 – 2.297	1.21	.23
GROUP[older] × TRANSITION MODE[into AP] × nTRIALSc	0.739	0.193	0.443 – 1.234	-1.16	.25
GROUP[older] × nTRIALSc	1.009	0.153	0.749 – 1.359	0.06	.95
TRANSITION MODE[into AP]	0.443	0.099	0.286 – 0.688	-3.63	**<.001**
**Random Effects**					
σ^2	3.29				
$\tau_{00\ subjID}$	0.95				
ICC	0.22				
N_{subjID}	42				
Observations	4614				
Marginal R^2 / Conditional R^2	0.131 / 0.325				
AIC BIC	2410.4 / 2468.3				

[$] *Intercept given on log scale.*

Supplementary Table 8 Results for beta GLMM to predict cumulative error rate [0<error rate/100 <1]

Type II Wald statistics

Predictors	X^2	*df*	*p*
GROUP	0.47	1	.49
TRANSITION MODE	4.909	1	.027
nTRIALSc	6.692	1	.01
GROUP × TRANSITION MODE	0.017	1	.90
GROUP × nTRIALSc	1.18	1	.28
TRANSITION MODE × nTRIALSc	1.023	1	.31
GROUP × TRANSITION MODE × nTRIALSc	1.053	1	.31

Parameter estimates based on references categories indicated

Predictors	*Estimates (β)*	*std. Error*	*CI*	*Statistic* (X^2)	*p*
(Intercept)	0.209	0.036	0.149 – 0.293	-9.07	**<.0001**
GROUP[young]	*Reference*				
TRANSITION MODE[into AP] × nTRIALSc	0.955	0.031	0.897 – 1.017	-1.44	.15
GROUP[older]	1.182	0.282	0.740 – 1.888	0.70	.48
nTRIALSc	1.067	0.024	1.021 – 1.116	2.87	**.004**
TRANSITION MODE[into IP]	*Reference*				
GROUP[older] × TRANSITION MODE[into AP]	0.992	0.047	0.904 – 1.088	-0.17	.87
GROUP[older] × TRANSITION MODE[into AP] × nTRIALSc	1.049	0.049	0.957 – 1.150	1.03	.31
GROUP[older] × nTRIALSc	0.952	0.031	0.892 – 1.015	-1.49	.14
TRANSITION MODE[into AP]	1.059	0.034	0.994 – 1.127	1.78	.08
**Random Effects**					
σ^2	0.27				
$\tau_{00\ subjID}$	0.59				
ICC	0.68				
N_{subjID}	42				
Observations	4227				
Marginal R^2 / Conditional R^2	0.010 / 0.687				
AIC / BIC	-7602.95 / -7539.5				

[$] *Parameter estimates' effect on cumulative error rate are given as change in ratio of proportion [exp(logit)].*

Supplementary Table 9 Results for gamma GLMM to predict transition latency

Type II Wald statistics

Predictors	X^2	*df*	*p*
GROUP	37.739	1	<.0001
TRANSITION MODE	8.917	1	<.003
nTRIALSc	3.949	1	.047
GROUP × TRANSITION MODE	0.331	1	.56
GROUP × nTRIALSc	0.13	1	.72
TRANSITION MODE × nTRIALSc	0.194	1	.66
GROUP × TRANSITION MODE × nTRIALSc	0.004	1	.95

Parameter estimates based on references categories indicated

Predictors	*Estimates (β)*	*std. Error*	*CI*	*Statistic (X^2)*	*p*
(Intercept)	568.73	25.06	521.68 – 620.03	143.98	**<.0001**
GROUP[young]	*Reference*				
TRANSITION MODE[into AP] × nTRIALSc	1.01	0.04	0.93 – 1.10	0.27	.79
GROUP[older]	1.38	0.08	1.22 – 1.55	5.22	**<.0001**
nTRIALSc	0.96	0.03	0.91 – 1.02	-1.37	.17
TRANSITION MODE[into IP]	*Reference*				
GROUP[older] × TRANSITION MODE[into AP]	1.03	0.06	0.92 – 1.16	0.57	.57
GROUP[older] × TRANSITION MODE[into AP] × nTRIALSc	1.00	0.06	0.89 – 1.13	0.06	.95
GROUP[older] × nTRIALSc	1.01	0.04	0.93 – 1.09	0.21	.83
TRANSITION MODE[into AP]	1.07	0.04	0.99 – 1.17	1.73	.08
**Random Effects**					
σ^2	0.20				
$\tau_{00\ subjID}$	0.00				
ICC	0.02				
N_{subjID}	42				
Observations	4614				
Marginal R^2 / Conditional R^2	0.128 / 0.147				
AIC / BIC	1339.98 / 1404.31				

[$] *Parameter estimates' effect on transition latency and CI given as change in ratio of proportion[exp(logit)].*

Supplementary Note 2 Thumb reaction time (tRT) – Operationalization and Statistical analysis

Thumb RT was optimally fit with a gamma distribution and therefore a GLMM (Gamma family with identity link) was fitted to predict tRT with GROUP, SIDE, and nTRIALSc. Factors GROUP (old, young), SIDE (left, right), and covariate nTRIALSc (trial number, centred) were entered as fixed effects. Random intercepts were fit on subject level. Trimming of tRT data resulted in discarding 1.8% of trials resulting in a total of 1176 tRT trials entering the analysis.

The model's total explanatory power is substantial (conditional R2 = 0.80) and the part related to the fixed effects alone (marginal R2) is of 1.0. The model's intercept, corresponding to GROUP = young, side= right, nTRIALSc = 0, is at 501.0ms (SE = 18.17, 95% CI [465.39, 536.61], $p < .0001$). Older participants were on average over 252ms slower as shown by the positive effect of GROUP[older] (beta = 252.70±16.38, 95% CI [220.59, 284.80], $p < .0001$). Overall, left side responses were more than 28ms slower compared to right side responses (beta = 28.89±9.21, 95% CI [10.84, 46.95], $p < .01$). A positive interaction effect of SIDE [left] on GROUP [older] (beta = 32.15±14.38, 95% CI [3.97, 60.34], $p < .05$) was followed up by contrasts estimated from marginal means, which revealed that both groups responded significantly slower with the left hand (OLDER| right – left: ΔEMM = -61.047± 13.909, 95% CI [-97.741, -24.353], z = -4.389, p<.0001; YOUNG| right – left: ΔEMM = -28.893± 9.213, 95% CI [-53.199, -4.587], z = -3.136, p <.01). Time across the experiment had neither a main effect on tRT (beta = -8.77±6.36, 95% CI [-21.23, 3.69], p >.1), nor was a relevant effect modulator of the other factors.

Supplementary Table 10 Results for gamma GLMM to predict thumb reaction time

Parameter estimates based on references categories indicated

Parameter	*Estimates (β)*	*std. Error*	*CI*	*Statistic (X^2)*	*p*
(Intercept)	501.00	18.17	465.39 – 536.61	27.58	<.0001
nTRIALSc	-8.77	6.36	-21.23 – 3.69	-1.38	.17
GROUP[young]	*Reference*				
GROUP[older] x nTRIALSc	-10.84	9.01	-28.51 – 6.82	-1.20	.23
GROUP[older] x SIDE[left]	32.15	14.38	3.97 – 60.34	2.24	**.03**
GROUP[older] x SIDE[left] x nTRIALSc	-3.36	11.67	-26.22 – 19.51	-0.29	.77
GROUP[older]	252.70	16.38	220.59 – 284.80	15.42	**<.0001**
SIDE[right]	*Reference*				
SIDE[left] x nTRIALSc	6.75	9.74	-12.34 – 25.85	0.69	.49
SIDE[left]	28.89	9.21	10.84 – 46.95	3.14	**.002**
Random Effects					
σ^2	0.07				
$\tau_{00\ subject}$	4496.13				
ICC	1.00				
$N_{subject}$	43				
Observations	1176				

Marginal R^2 / Conditional R^2	0.80 / 1.0
AIC / BIC	15437.25 /15487.95

Supplementary material for the EEG data analysis

Supplementary Figure 4 Statistical results of spectral power changes.

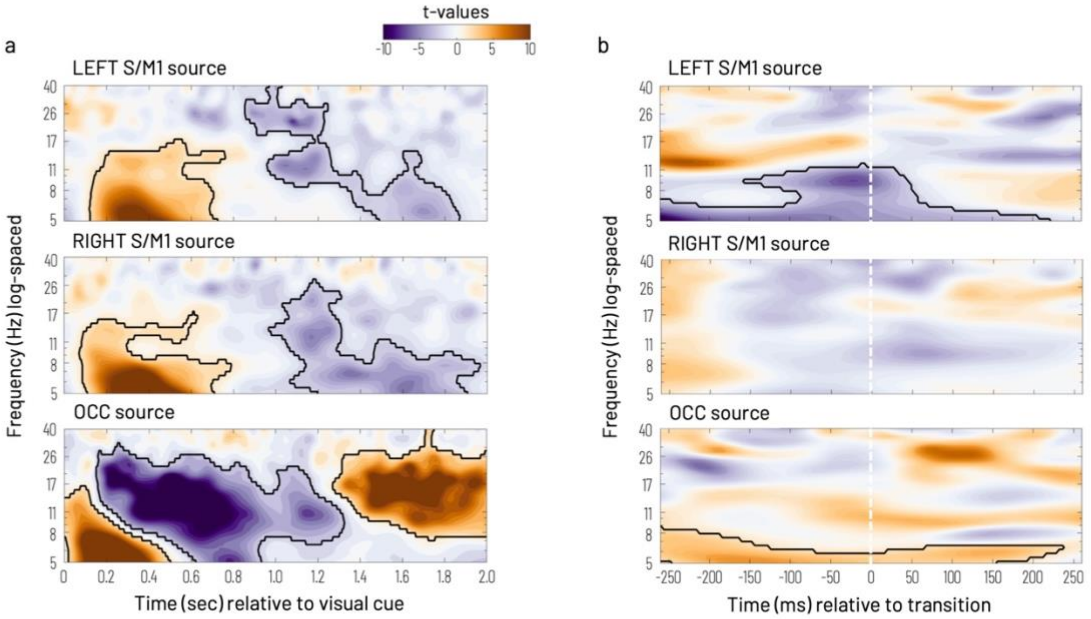

Supplementary Figure 4 a) Statistical results of cluster corrected permutation test for significant power changes from baseline, pooled over age groups and transition modes. *Time (in sec) is presented relative to visual cue onset (at 0 sec.). Orange color shading indicates significant power increase and purple color shading indicates significant power decrease with respect to the baseline period [-500 to -200ms relative to cue onset]. Black lines highlight significant frequency-by-time clusters (2-sided t-test, permutation-based cluster correction).* ***b) Statistical results of cluster corrected permutation test for significant power changes for group contrast (OLDER vs. YOUNG) for the transition mode difference (IP - AP).*** *Orange color shading indicates significant power increase and purple color shading indicates significant power decrease for the OLDER relative to the YOUNG. Black lines highlight significant frequency-by-time clusters (2-sided t-test, permutation-based cluster correction at $p<.05$). Time (in ms) is presented relative to the response (white vertical dashed line at 0ms). Zooming into the time window ±260ms around the individual median transition latency for the analysis of the effect of transition mode and its modulation by factor age group, i.e. running a two samples t-test on the contrast $[IP-AP]_{YOUNG} – [IP-AP]_{OLDER}$, revealed specific time and frequency clusters reaching level of significance ($p<.05$, 2-sided) for the individual sources.*

.

Supplementary Figure 5 Stimulus-locked analysis of ISPC modulation.

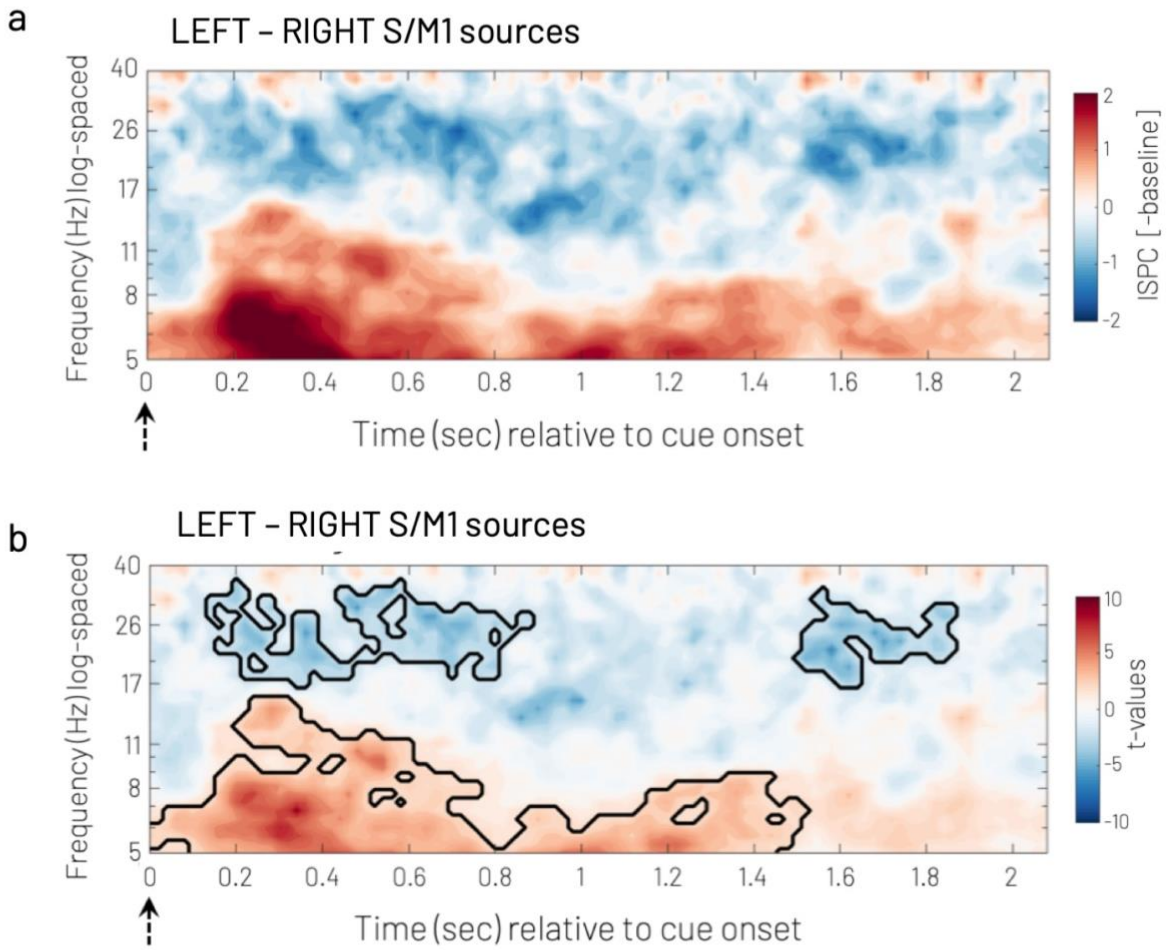

Supplementary Figure 5a) ***Baseline subtracted ISPC.*** *Time and frequency distribution of change in connectivity to baseline (-500 to -200ms before visual cue onset) for left S/M1 – right S/M1 sources pooled over age groups and transition modes. Red shading indicates a relative increase in connectivity, blue shading indicates a relative decrease. Black arrow at time 0ms indicates visual start cue.* ***b) Statistical results of time and frequency resolved modulation of connectivity*** *relative to baseline (ISPC baseline subtracted). Clusters of significant connectivity change (corrected t-values shown for p<.05, 2-tailed) are highlighted with black lines. Colour coding of direction of effects as in a). The analysis of connectivity modulation revealed significant time × frequency clusters when pooled over age groups and transition modes. The left – right S/M1 connection showed a distributed connectivity increase in the theta, alpha, and mu range from cue onset until 1500ms. Connectivity increased most prominently around 300ms in the theta and low alpha range. Around this time, there was also increased connectivity in the upper alpha/mu and low beta range, which was not visible later in the time window of interest. Left – right S/M1 connectivity significantly decreased in the beta spectrum (17 – 40 Hz) around 200ms until 900ms following the visual cue onset. A second cluster between 1500 until 1900ms showed also a connectivity decrease in the beta frequency band.*

Supplementary results for phase angle differences

Supplementary Figure 1 – Phase angle differences pooled over transition modes within age group

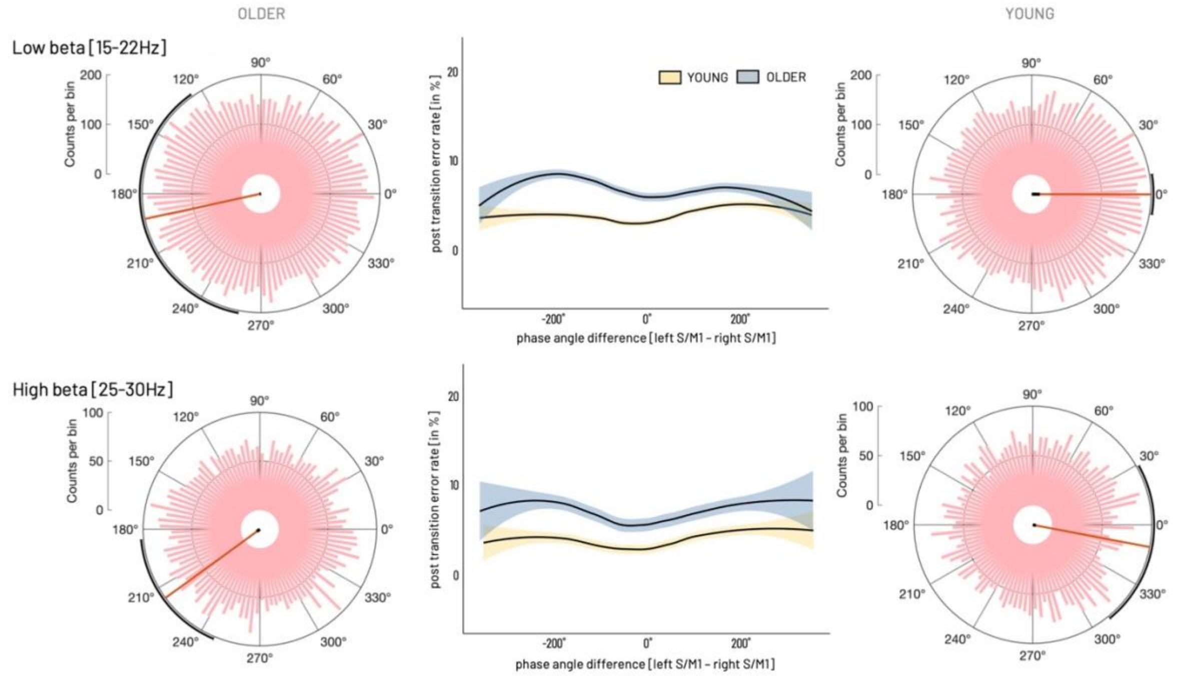

Supplementary Figure 1 *Phase angle differences pooled over transition modes within group for low beta (15-22Hz, top row) and high beta (25-30Hz, bottom row) frequency bands. Rose plots show histogram of binned phase angle differences with mean direction (red line) and 95% CI (black circumference) for significant non-uniformity of distribution for older (left) and young group (right). Line plots depict circular correlation of phase angle differences with post transition error [in %] shown separately for age groups (yellow – young, blue – older). Both age groups show lower errors after transition for 0˚ (or 360˚, respectively) phase lag between left and right S/M1 at the time of transition.*

Supplementary Note 3 Rayleigh test for distribution of phase angle differences between left S/M1 and right S/M1 sources at time of transition for transition modes pooled over age groups.

When pooled over age groups, non-uniformity of phase angle-differences between left and right S/M1 sources was shown for transitions into IP for the low beta band only (15-22Hz: z = 11.21, p_{FDR} = 4.99e-05), whereas for transitions into AP this was the case for both, the low and the high beta band (15-22Hz: z = 12.21, p_{FDR} = 2.45e-05; 25-30Hz: z = 4.09, p_{FDR} =.05).

Supplementary Table 11 Rayleigh test for distribution of phase angle differences between left S/M1 and right S/M1 sources at time of transition accounting for GABA+ concentration relative to within group median

GABA+ relative to group median	group	frequency range (Hz)	z	p_{FDR}
low	YOUNG	15 - 22	25.16	1.08e-10***
low	YOUNG	25 - 30	2.87	0.21
low	OLDER	15 - 22	2.66	0.24
low	OLDER	25 - 30	6.51	0.008*
high	YOUNG	15 - 22	47.58	2.53e-20***
high	YOUNG	25 - 30	6.38	0.008*
high	OLDER	15 - 22	1.42	0.75
high	OLDER	25 - 30	5.56	0.02*

Supplementary Table 12 Rayleigh test for distribution of phase angle differences between left S/M1 and right S/M1 sources at baseline [START CUE – 300ms] accounting for GABA+ concentration relative to within group median

GABA+ relative to group median	group	frequency range (Hz)	z	p_{FDR}
low	YOUNG	15 - 22	55.51	8.85e-24
low	YOUNG	25 - 30	6.60	0.006
low	OLDER	15 - 22	47.29	2.05e-20
low	OLDER	25 - 30	8.29	0.001
high	YOUNG	15 - 22	81.41	6.86e-35
high	YOUNG	25 - 30	5.34	0.02
high	OLDER	15 - 22	12.95	1.47e-05
high	OLDER	25 - 30	2.27	n.s. (0.32)

Supplementary Table 13 2-way ANOVA testing GROUP (older vs. young) x GABA(low vs. high) for mean phase angle difference between left S/M1 and right S/M1 sources at baseline [START CUE – 300ms]

Frequency range	Source	d.f.	X^2	P-Value
15-22Hz	GROUP	2	71.09	3.33e-16
	GABA+	2	47.87	4.04e-11
	Interaction	1	87.38	0
25-30Hz	GROUP	2	5.10	0.05
	GABA+	2	10.25	0.006
	Interaction	1	8.40	0.004

Supplementary Table 14 Circular-linear correlation phase angle difference ~ subsequent error

GABA+ relative to group median	group	frequency range (Hz)	rho	p_{FDR}
low	YOUNG	15 - 22	0.05	0.0001
low	YOUNG	25 - 30	0.01	n.s. (2.40)
low	OLDER	15 - 22	0.10	0
low	OLDER	25 - 30	0.08	3.61e-06
high	YOUNG	15 - 22	0.11	0
high	YOUNG	25 - 30	0.10	5.28e-08
high	OLDER	15 - 22	0.06	6.10e-07
high	OLDER	25 - 30	0.02	n.s. (1.09)

Supplementary Figure 2 Association between band-specific left - right S/M1 phase difference at BASELINE [START CUE – 300ms] and subsequent performance pooled over transition conditions (group average of single trial baseline, error represents subsequent trial following the START CUE)

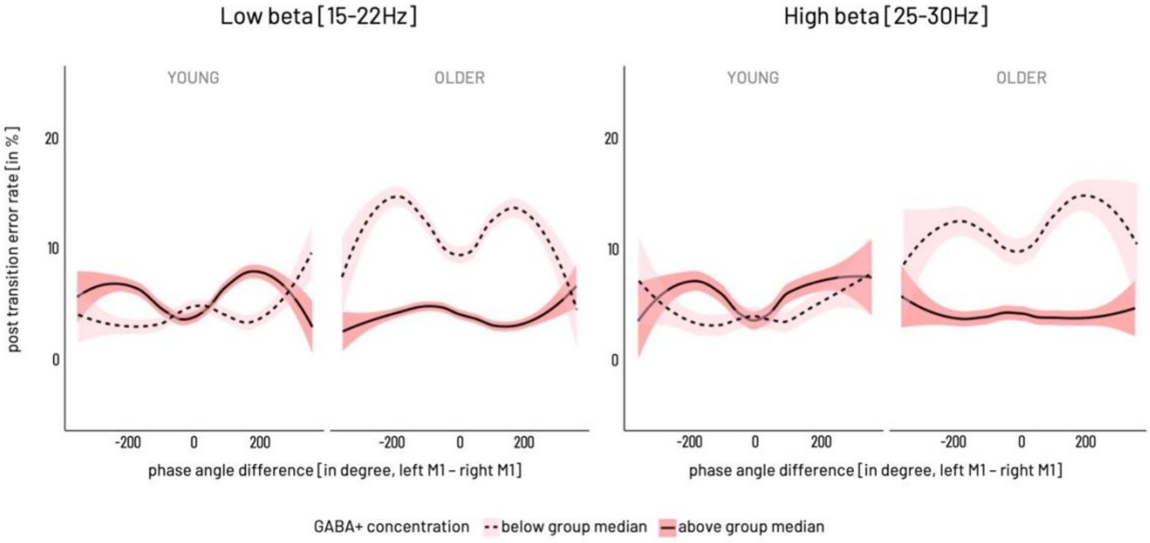

Supplementary Figure 2 a) *Group averages of phase angle difference between left and right S/M1 sources for low beta frequency range.* ***b)*** *Group averages for phase angle difference between left and right S/M1 sources for high beta frequency range.*

Supplementary Table 15 Comparison left -right S/M1 versus OCC-S/M1

Rayleigh test for non-uniformity of phase angle difference distribution

OCC- S/M1

Interaction between	GABA+ relative to group median	group	frequency range (Hz)	z	p_{FDR}
OCC-LEFT S/M1	low	YOUNG	15 - 22	16.88	1.73e-06
	low	YOUNG	25 - 30	0.73	1.81
	low	OLDER	15 - 22	5.27	0.05
	low	OLDER	25 - 30	2.27	0.68
	high	YOUNG	15 - 22	1.30	1.30
	high	YOUNG	25 - 30	6.21	0.023
	high	OLDER	15 - 22	0.72	1.81
	high	OLDER	25 - 30	0.34	2.42
OCC - RIGHT S/M1	low	YOUNG	15 - 22	1.0	1.37
	low	YOUNG	25 - 30	0.11	2.79
	low	OLDER	15 - 22	8.96	0.002
	low	OLDER	25 - 30	6.17	0.02
	high	YOUNG	15 - 22	4.14	0.07
	high	YOUNG	25 - 30	11.21	0.0003
	high	OLDER	15 - 22	0.83	1.47
	high	OLDER	25 - 30	5.19	0.03

Supplementary Table 16 2-way ANOVA testing mean direction CONNECT (LEFT S/M1 -RIGHT S/M1 vs. OCC-LEFT S/M1) x group (older vs young)

Frequency range	Source	d.f.	X^2	P-Value

15-22Hz	CONNECT	2	10.78	0.005
	GROUP	2	50.07	1.34e-11
	Interaction	1	51.73	6.36e-13
25-30Hz	CONNECT	2	0.70	0.70
	GROUP	2	7.15	0.03
	Interaction	1	15.40	8.72e-05

Supplementary Table 17 2-way ANOVA testing mean direction CONNECT (LEFT S/M1 - RIGHT S/M1 vs. OCC-RIGHT S/M1) x GROUP (older vs young)
15-22Hz

Frequency range	Source	d.f.	X^2	P-Value
15-22Hz	CONNECT	2	13.01	0.002
	GROUP	2	43.67	3.30e-10
	Interaction	1	56.10	6.90e-14
25-30Hz	CONNECT	2	6.76	0.03
	GROUP	2	41.65	9.04e-10
	Interaction	1	9.67	0.002

Supplementary Table 18 Circular-linear correlation phase angle difference ~ subsequent error

Interaction between	GABA+ relative to group median	group	frequency range (Hz)	rho	p_{FDR}
OCC – LEFT S/M1	low	YOUNG	15 - 22	0.024	ns (0.44)
	low	YOUNG	25 - 30	0.04	ns (0.36)
	low	OLDER	15 - 22	0.03	ns (0.14)
	low	OLDER	25 - 30	0.04	ns (0.21)
	high	YOUNG	15 - 22	0.03	ns (0.36)
	high	YOUNG	25 - 30	0.04	ns (0.43)
	high	OLDER	15 - 22	0.01	ns (1.88)
	high	OLDER	25 - 30	0.05	ns (0.14)
OCC – RIGHT S/M1	low	YOUNG	15 - 22	0.02	ns (1.46)
	low	YOUNG	25 - 30	0.05	ns (0.62)
	low	OLDER	15 - 22	0.02	ns (0.69)
	low	OLDER	25 - 30	0.01	ns (2.87)
	high	YOUNG	15 - 22	0.02	ns (1.19)
	high	YOUNG	25 - 30	0.02	ns (2.02)
	high	OLDER	15 - 22	0.01	ns (2.02)
	high	OLDER	25 - 30	0.02	ns (1.98)

Supplementary Figure 3 Association between band-specific OCC-M1 phase difference at time of transition and subsequent performance pooled over transition conditions

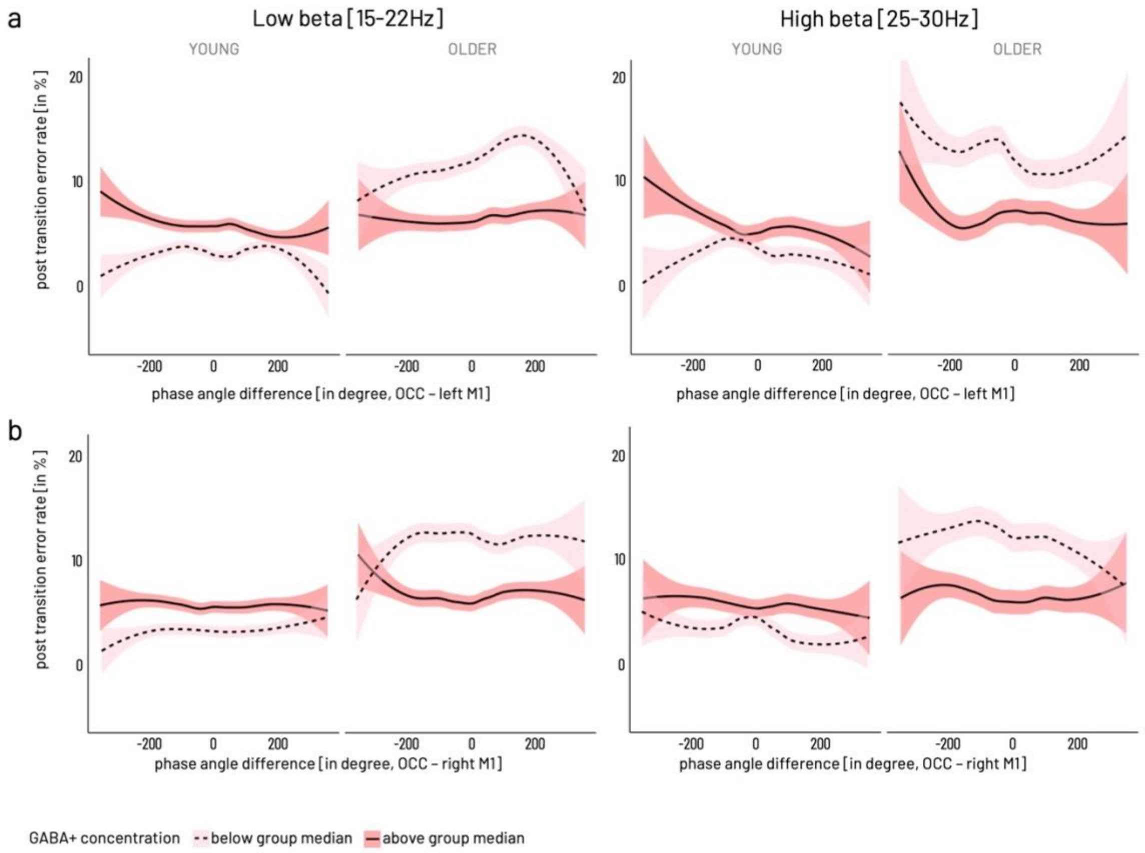

Supplementary Figure 3 a) *Phase angle difference between occipital and left S/M1 source (OCC-left S/M1).* ***b)*** *Phase angle difference between occipital and right S/M1 source (OCC-right S/M1).*

Supplementary results for the Bayesian moderated mediation analyses

Supplementary Table 19 Regression coefficients of Bayesian moderated mediation models

DV	Mediator	LEFT S/M1 GABA+			RIGHT S/M1 GABA+		
	Time Window	PRE	PERI	POST	PRE	PERI	POST
	Frequency band of IV	high beta	low beta	high alpha / mu	high beta	low beta	high alpha / mu
error rate	τ	0.032<br>[-0.057, 0.121]<br>71.5%	**0.087<br>[0.031, 0.141]<br>99.5%****	**0.104<br>[0.06, 0.145]<br>100%****	0.055<br>[-0.041, 0.151]<br>81.9%	**0.084<br>[0.026, 0.136]<br>99.2%***	**0.104<br>[0.061, 0.146]<br>99.9%****
	α	0.073<br>[-0.216, 0.361]<br>65.7%	**0.447<br>[0.25, 0.65]<br>100%****	-0.007<br>[-0.157, 0.128]<br>53.1%	**-0.507<br>[-0.897, -0.123]<br>98.2%***	**0.381<br>[0.164, 0.601]<br>99.7%****	0.148<br>[-0.044, 0.345]<br>88.9%
	β	0.032<br>[-0.028, 0.092]<br>79.8%	0.028<br>[-0.019, 0.072]<br>82.9%	0.041<br>[-0.021, 0.099]<br>85.2%	-0.035<br>[-0.09, 0.021]<br>84.5%	**-0.049<br>[-0.086, -0.011]<br>98.2%****	-0.05<br>[-0.1, 0.001]<br>94.4%
	α × β	0.001<br>[-0.011, 0.017]<br>59.6%	0.011<br>[-0.01, 0.033]<br>82.9%	0.0<br>[-0.008, 0.007]<br>52.4%	0.015<br>[-0.012, 0.052]<br>83.3%	**-0.017<br>[-0.036, -0.001]<br>97.9%***	-0.006<br>[-0.02, 0.005]<br>84.5%
	τ’	0.029<br>[-0.059, 0.117]<br>70.2%	**0.075<br>[0.017, 0.131]<br>98.1%***	**0.104<br>[0.062, 0.146]<br>100%****	0.037<br>[-0.056, 0.13]<br>73.6%	**0.102<br>[0.048, 0.157]<br>99.8%****	**0.112<br>[0.068, 0.151]<br>100%****
transition latency	τ	-0.005<br>[-0.172, 0.159]<br>52.0%	**0.186<br>[0.093, 0.281]<br>99.9%****	**0.181<br>[0.098, 0.27]<br>100%****	-0.082<br>[-0.252, 0.089]<br>78.2%	**0.138<br>[0.04, 0.235]<br>98.8%***	**0.143<br>[0.055, 0.232]<br>99.3%***
	α	0.075<br>[-0.214, 0.356]<br>66.2%	**0.448<br>[0.253, 0.653]<br>100%****	-0.007<br>[-0.154, 0.129]<br>53.2%	**-0.506<br>[-0.897, -0.125]<br>98.2%***	**0.381<br>[0.16, 0.595]<br>99.7%****	0.148<br>[-0.041, 0.34]<br>89.4%
	β	-0.034<br>[-0.163, 0.085]<br>67.3%	-0.048<br>[-0.136, 0.047]<br>79.8%	-0.003<br>[-0.123, 0.116]<br>51.6%	0.049<br>[-0.071, 0.168]<br>74.9%	0.052<br>[-0.026, 0.133]<br>85.3%	0.055<br>[-0.055, 0.162]<br>78.5%
	α × β	-0.001<br>[-0.028, 0.021]<br>55.1%	-0.02<br>[-0.066, 0.02]<br>79.8%	0<br>[-0.011, 0.009]<br>50.0%	-0.02<br>[-0.096, 0.038]<br>74.1%	0.018<br>[-0.013, 0.053]<br>85.1%	0.005<br>[-0.011, 0.033]<br>72.5%
	τ’	-0.003<br>[-0.161, 0.165]<br>50.9%	**0.208<br>[0.109, 0.303]<br>100%****	**0.181<br>[0.093, 0.264]<br>100%****	-0.057<br>[-0.225, 0.115]<br>70.8%	**0.118<br>[0.026, 0.217]<br>97.6%***	**0.135<br>[0.05, 0.221]<br>99.3%***

*Regression coefficient of model paths given as median [89% HDI], pd (in %). Asterisks indicate approximate 2-tailed p-value ° p<.1, * p<.05, **p<.01*

Supplementary References

1. Marczynski, T. J. GABAergic Deafferentation Hypothesis of Brain Aging and Alzheimer's Disease Revisited. *Brain Research Bulletin* **45**, 341–379 (1998).
2. Scimemi, A. Structure, function, and plasticity of GABA transporters. *Front. Cell. Neurosci.* **8**, (2014).
3. Cuypers, K. *et al.* Age-related GABAergic differences in the primary sensorimotor cortex: A multimodal approach combining PET, MRS and TMS. *NeuroImage* **226**, 117536 (2021).
