# Supplementary material for: The interaction between endogenous GABA, functional connectivity, and behavioral flexibility is critically altered with advanced age
Source: Commun Biol. 2022 May 6;5:426. doi: 10.1038/s42003-022-03378-w (PMC9076638; doi:10.1038/s42003-022-03378-w)
Supplement: Supplementary file 3 — Reporting Summary [file 42003_2022_3378_MOESM3_ESM.pdf]

nature research

Corresponding author(s): Kirstin-Friederike Heise
Department of Movement Sciences
KU Leuven
Tervuursevest 101
3001 Leuven, Belgium

Last updated by author(s): Mar 4, 2022

Reporting Summary

Nature Research wishes to improve the reproducibility of the work that we publish. This form provides structure for consistency and transparency in reporting. For further information on Nature Research policies, see our Editorial Policies and the Editorial Policy Checklist.

Statistics

For all statistical analyses, confirm that the following items are present in the figure legend, table legend, main text, or Methods section.

n/a	Confirmed	
☐	☒	The exact sample size (*n*) for each experimental group/condition, given as a discrete number and unit of measurement
☐	☒	A statement on whether measurements were taken from distinct samples or whether the same sample was measured repeatedly
☐	☒	The statistical test(s) used AND whether they are one- or two-sided<br>*Only common tests should be described solely by name; describe more complex techniques in the Methods section.*
☐	☒	A description of all covariates tested
☐	☒	A description of any assumptions or corrections, such as tests of normality and adjustment for multiple comparisons
☐	☒	A full description of the statistical parameters including central tendency (e.g. means) or other basic estimates (e.g. regression coefficient) AND variation (e.g. standard deviation) or associated estimates of uncertainty (e.g. confidence intervals)
☐	☒	For null hypothesis testing, the test statistic (e.g. *F*, *t*, *r*) with confidence intervals, effect sizes, degrees of freedom and *P* value noted<br>*Give P values as exact values whenever suitable.*
☐	☒	For Bayesian analysis, information on the choice of priors and Markov chain Monte Carlo settings
☒	☐	For hierarchical and complex designs, identification of the appropriate level for tests and full reporting of outcomes
☐	☒	Estimates of effect sizes (e.g. Cohen's *d*, Pearson's *r*), indicating how they were calculated

Our web collection on statistics for biologists contains articles on many of the points above.

Software and code

Policy information about availability of computer code

Data collection	GABA-MRS data were acquired using the Mescher–Garwood point resolved spectroscopy (MEGA-PRESS) sequence.<br>Visual and auditory stimuli of the behavioral paradigm were programmed in LabVIEW 2016 (National Instruments, Austin/TX, USA), also used for behavioral data collection. This code is available from the corresponding author upon request.<br>EEG data was acquired with BrainVision Recorder, version 1.21.0004, BrainProducts GmbH, Gilching/Germany.
Data analysis	MRS data were preprocessed and analysed with the Gannet software 3.0 toolkit implemented in Matlab (2018b, MathWorks, Natick, MA, USA). To adjust GABA concentration for heterogeneity in voxel tissue composition, MRS voxels co-registered to the high-resolution anatomical image were segmented into three different tissue classes, namely gray matter (GM), white matter (WM), and cerebrospinal fluid (CSF), with SPM 12 (http://www.fil.ion.ucl.ac.uk/spm/software/spm12/), also implemented in Matlab.<br>Behavioral data was preprocessed with custom-written scripts in Matlab, available from the corresponding author upon request.<br>EEG data (pre-) processing and analyses were performed using functions from the EEGLAB toolbox version 2019.0, the Fieldtrip toolbox version 20190419, and customised Matlab functions (Matlab 2018b, MathWorks, Natick, MA, USA).<br>For the forward solution, an individual head model was created for each participant based on the same high-resolution structural MR image as used for the MRS analysis and 3D locations of the electrodes, registered with an optical infrared-camera based (NDI, Ontario, Canada) neuronavigation system (xensor™, ANT Neuro, Enschede, Netherlands).<br>For the individual geometrical description of the head (mesh), the anatomical image was segmented into 12 tissue classes (skin, eyes, muscle, fat, spongy bone, compact bone, cortical gray matter, cerebellar gray matter, cortical white matter, cerebellar white matter, cerebrospinal fluid and brain stem), based on the MIMA model 66 using SPM12 (http://www.fil.ion.ucl.ac.uk/spm/software/spm12/).<br>The volume conductor model was constructed based on a whole-head finite element model 73 using the SimBio toolbox (https://www.mrt.uni-jena.de/simbio) implemented in FieldTrip. For inverse-modelling of source activity, we used the exact low-resolution brain electromagnetic tomography (eLoreta) algorithm.<br>For the statistical analyses, all (generalized) linear-mixed effects models were run in R for statistical computing (R version 4.0.2 (2020-06-22),

Platform: x86_64-apple-darwin17.0 (64-bit) under macOS Mojave 10.14.6; R Core Team (2020). R: A language and environment for statistical computing. R Foundation for Statistical Computing, Vienna, Austria. URL https://www.R-project.org/). The following libraries were used to implement the generalized linear mixed-effects models including pairwise testing of contrasts of parameter estimates for respective factor levels: lme4, nlme, easystats, ggeffects, emmeans,parameters, modelbased, effectsize, performance.
Posterior distributions for multivariate models were obtained using Hamiltonian Monte-Carlo algorithm using Stan implemented for R with brms, and rstanarm packages. Circular statistics and visual representations were performed with CircStat and CircHist (https://github.com/zifredder/CircHist) toolboxes implemented for Matlab 2018b and R package circular (version 0.4-93).

For manuscripts utilizing custom algorithms or software that are central to the research but not yet described in published literature, software must be made available to editors and reviewers. We strongly encourage code deposition in a community repository (e.g. GitHub). See the Nature Research guidelines for submitting code & software for further information.

Data

Policy information about availability of data

All manuscripts must include a data availability statement. This statement should provide the following information, where applicable:

- Accession codes, unique identifiers, or web links for publicly available datasets
- A list of figures that have associated raw data
- A description of any restrictions on data availability

General source data are not publicly available due to European legal restrictions compromising the research participants' privacy and consent. Source data to reproduce results given in figures 2-7 are provided under [https://figshare.com/s/c2df37a0f7f68b9d208c]. Additional code to reproduce the experimental set-up and further results is available from the corresponding author [KFH].

Field-specific reporting

Please select the one below that is the best fit for your research. If you are not sure, read the appropriate sections before making your selection.

☐ Life sciences ☒ Behavioural & social sciences ☐ Ecological, evolutionary & environmental sciences

For a reference copy of the document with all sections, see nature.com/documents/nr-reporting-summary-flat.pdf

Behavioural & social sciences study design

All studies must disclose on these points even when the disclosure is negative.

Study description	quantitative experimental cross-sectional
Research sample	44 volunteers (older group N = 22, age range 62-82 years of age; young group N = 22, age range 21 – 27 years of age).
Sampling strategy	No statistical method was performed for an a priori sample size calculation; rather, we based reasoning for the selected sample size on numbers chosen in previous multimodal work.
Data collection	MRI/MRS, behavioral, and EEG data were digitized. Participant characteristics and personal information were recorded with pen and paper forms. Additional information concerning experimental procedures were documented with pen on standardized experimental forms.
Timing	Dec, 1st, 2018 - March 30th, 2019
Data exclusions	MRS data acquisition and reporting was done following the Magnetic Resonance Spectroscopy quality assessment tool (MRS-Q). In two cases (one older, one young), the data of the right M1 were excluded from further analysis due to motion artifacts and insufficient model fit.<br>All available behavioral data were analyzed based on the time window of interest (between cue and 2000ms). No 'outlier exclusion' was performed.<br>EEG data analysis consisted of a multi-step procedure involving manual/visual inspection and semi-automated processing steps to preserve as much signal as possible and eliminate noise (eg. from muscle contraction) as necessary.
Non-participation	One young participant dropped out after the MRI data acquisition for personal reasons unrelated to the study. MRI, EEG, and behavioral data were thus collected in 21 young (10 women) and 22 older (11 women) participants. Due to technical problems, the EEG of one young participant had to be excluded, yielding different numbers of data sets included into the analysis for GABA, behavioral, and EEG analysis.
Randomization	n/a

Reporting for specific materials, systems and methods

We require information from authors about some types of materials, experimental systems and methods used in many studies. Here, indicate whether each material, system or method listed is relevant to your study. If you are not sure if a list item applies to your research, read the appropriate section before selecting a response.

Materials & experimental systems

n/a	Involved in the study
☒	☐ Antibodies
☒	☐ Eukaryotic cell lines
☒	☐ Palaeontology and archaeology
☒	☐ Animals and other organisms
☐	☒ Human research participants
☒	☐ Clinical data
☒	☐ Dual use research of concern

Methods

n/a	Involved in the study
☒	☐ ChIP-seq
☒	☐ Flow cytometry
☐	☒ MRI-based neuroimaging

Human research participants

Policy information about studies involving human research participants

Population characteristics	see above
Recruitment	Participants were recruited through local advertisements and were screened for in- and exclusion criteria (e.g., contraindication against MRI imaging, intake of neuroactive medication).
Ethics oversight	Medical Ethical Committee of the KU/UZ Leuven, Belgiun, National registration number of the approved protocol B322201628182

Note that full information on the approval of the study protocol must also be provided in the manuscript.

Magnetic resonance imaging

Experimental design

Design type	resting-state acquisition of MR-spectroscopy data
Design specifications	n/a
Behavioral performance measures	n/a

Acquisition

Imaging type(s)	structural and MRI spectroscopy
Field strength	3T
Sequence & imaging parameters	3D high-resolution T1-weighted structural image (repetition time = 9.5 ms; echo time = 4.6 ms; voxel size = 0.98 x 0.98 x 1.2 mm3; field of view = 250 x 250 x 222 mm3; 185 coronal slices) ;<br>GABA-MRS data were acquired using the Mescher–Garwood point resolved spectroscopy (MEGA-PRESS) sequence, with parameters resembling those of previous work 15–17; 14ms sinc-Gaussian editing pulses applied at an offset of 1.9 ppm in the ON experiment and 7.46 ppm in the OFF experiment, TR = 2000ms, TE = 68ms, 2000 Hz spectral bandwidth, MOIST water suppression, 320 averages, scan duration of 11 minutes, 12 seconds]. Sixteen water-unsuppressed averages were acquired from the same voxel. These scan parameters were identical for all three voxels (each 30x30x30mm in size).
Area of acquisition	T1 whole brain;<br>MRS voxels positioned based on the T1-weighted image. For the left and right sensorimotor voxels, this was centered above the hand knob area and rotated in the coronal and sagittal planes to align with the cortical surface of the brain. The occipital voxel was medially centered over the interhemispheric fissure, with the inferior boundary of the voxel aligned in parallel to the Tentorium cerebelli to cover left and right occipital lobes symmetrically.

Diffusion MRI ☐ Used ☒ Not used

Preprocessing

Preprocessing software	MRS data were analyzed with the Gannet software 3.0 toolkit 55. Individual frequency domain spectra were frequency- and phase-corrected using spectral registration 56 and filtered with a 3Hz exponential line broadening. Individual ON and OFF spectra were averaged and subtracted, yielding an edited difference spectrum, which was modelled at 3ppm with a single Gaussian peak and a 5-parameter Gaussian model. The unsuppressed water signal serving as the reference compound 57, was fit with a Gaussian-Lorentzian model. The integrals of the modelled data were then used to quantify the uncorrected GABA levels. To adjust GABA+ levels for heterogeneity in voxel tissue composition, MRS voxels co-registered to the high-resolution anatomical image were segmented into three different tissue classes, namely gray matter (GM), white matter (WM), and cerebrospinal fluid (CSF), with SPM 12 (http://www.fil.ion.ucl.ac.uk/spm/software/spm12/). The resulting voxel

	compositions were used to extract tissue-corrected GABA+ following the assumptions that GABA+ levels are negligible in CSF and twice as high in GM relative to WM, accounting for tissue-specific relaxation and water visibility values.
Normalization	GABA+ levels were normalized to the average voxel composition within each age group after outlier removal (as specified above).
Normalization template	registration to MNI space for cross-modality analysis (MRS - EEG source-space)
Noise and artifact removal	n/a
Volume censoring	n/a

Statistical modeling & inference

Model type and settings	n/a
Effect(s) tested	n/a

Specify type of analysis: ☐ Whole brain ☒ ROI-based ☐ Both

Anatomical location(s)	Localization of left/right primary motor cortex voxel based on identification hand knob as anatomical landmark following Yousry, T. A. et al. Localization of the motor hand area to a knob on the precentral gyrus. A new landmark. Brain 120, 141-157 (1997).<br>Anatomical identification of occipital cortex voxel placement following Baumgarten, T. J. et al. Beta Peak Frequencies at Rest Correlate with Endogenous GABA+/Cr Concentrations in Sensorimotor Cortex Areas. PLoS One 11, e0156829 (2016).

Statistic type for inference<br>(See Eklund et al. 2016)	n/a
Correction	n/a

Models & analysis

n/a	Involved in the study
☒	☐ Functional and/or effective connectivity
☒	☐ Graph analysis
☒	☐ Multivariate modeling or predictive analysis
